# Induction of Pluripotency in Adult Equine Fibroblasts without c-MYC

**DOI:** 10.1155/2012/429160

**Published:** 2012-03-19

**Authors:** Khodadad Khodadadi, Huseyin Sumer, Maryam Pashaiasl, Susan Lim, Mark Williamson, Paul J. Verma

**Affiliations:** ^1^Centre for Reproduction and Development, Monash Institute of Medical Research, Monash University, Clayton, VIC 3800, Australia; ^2^Faculty of Medicine, Tabriz University of Medical Sciences, Tabriz 51666-14766, Iran; ^3^Stem Cell Technologies i (SCTi), Gleneagles Medical Centre, Singapore 258499; ^4^Gribbles Veterinary, Clayton, VIC 3168, Australia; ^5^South Australian Research Institute (SARDI), Turretfield Research Centre, Rosedale, SA 5350, Australia

## Abstract

Despite tremendous efforts on isolation of pluripotent equine embryonic stem (ES) cells, to date there are few reports about successful isolation of ESCs and no report of *in vivo* differentiation of this important companion species. We report the induction of pluripotency in adult equine fibroblasts via retroviral transduction with three transcription factors using *OCT4, SOX2*, and *KLF4* in the absence of c-MYC. The cell lines were maintained beyond 27 passages (more than 11 months) and characterized. The equine iPS (EiPS) cells stained positive for alkaline phosphatase by histochemical staining and expressed OCT4, NANOG, SSEA1, and SSEA4. Gene expression analysis of the cells showed the expression of *OCT4, SOX2 NANOG*, and *STAT3*. The cell lines retained a euploid chromosome count of 64 after long-term culture cryopreservation. The EiPS demonstrated differentiation capacity for the three embryonic germ layers both *in vitro* by embryoid bodies (EBs) formation and *in vivo* by teratoma formation. In conclusion, we report the derivation of iPS cells from equine adult fibroblasts and long-term maintenance using either of the three reprogramming factors.

## 1. Introduction

Cartilage and tendon injuries are common features of tissue damage in both humans and horses. These two tissues have a poor vascular system with low mitotic ability and therefore a limited ability for self-repair. The reduced performance and reinjury create considerable attention for treatments [1].

Adult mesenchymal stem cells (MSCs), embryonic stem cells (ESCs), and reprogrammed somatic cells such as induced pluripotent stem (iPS) cells can provide potential sources of cells for treatment of cartilage and tendon injuries.

MSCs can be isolated from different sources such as bone marrow aspirates [2], umbilical cord [3], and adipose tissue [4] and have the ability to differentiate into different cell types such as muscle, cartilage, and bone [5–7]. MSCs have been used for treatment of cartilage injuries in equines and humans. Although there were the early improvements in cartilage injuries, no significant or long-term recovery could be observed [8, 9]. In addition, MSCs are limited in bone marrow aspirates and need to be cultured after isolation for at least 4 weeks and have limited *in vitro* differentiation potential compared with ESCs [1, 10]. 

ES cells can overcome this limitation, as they can provide an inexhaustible supply of cell derivatives of all three germ layers. Despite tremendous efforts on isolation of ESCs, to date there are a few reports on isolation of equine ESCs, which had limited success and no investigation of *in vivo *differentiation of the isolated cells [11, 12]. Isolation of equine ESCs is difficult due to the shortage of oocytes and embryos, as well as complexity associated with oocyte collection, maturation, IVF, and *in vitro* culture in this species [13]. Even if ES cells can be successfully derived, a subsequent problem is the anticipated immune rejection of the derivatives of ES cells by the recipient due to incompatibility of the major histocompatibility complex (MHC) antigens because of differences in genomic DNA compared with that of the recipient [14].

There are alternative methods to produce autologous cells lines via reprogramming of adult somatic cells to the pluripotent states such as somatic cell nuclear transfer (SCNT) [15, 16] and induced pluripotent stem (iPS) cells [17]; however, limitation with derivation of equine ESCs extend to SCNET-ESC isolation as well.

Takahashi and Yamanaka [17] reported the generation of pluripotent cells from adult mouse fibroblast following retroviral-mediated transduction of four transcription factors, *OCT4, SOX2, c-MYC*, and *KLF4*. A number of studies have shown that iPS cells are similar to ESCs in morphology and epigenetic status, expression of pluripotent markers, and ability to differentiate into derivatives of all three embryonic germ layers both *in vivo* and *in vitro* and contribute to the germ-line in chimeric mice confirming their true pluripotency [17–19]. Therefore, these cells could have therapeutic application in both human and animals.

Pluripotency has been induced in somatic cells from human [20], primate [21], rat [22, 23] pigs [24–27], sheep [21], and cattle [28].

More recently, the generation of equine iPS cell lines from fetal fibroblasts using transposon-based delivery of four factors has been reported [29]. In this study, we report the generations of equine-induced pluripotent stem (EiPS) cells by retroviral-mediated transduction of adult equine fibroblasts using three transcription factors: *OCT4, SOX2, *and* KLF4*, *(OSK) *without the protooncogene* c-MYC, *and the pluripotent characteristics of the resulting EiPS cells have been demonstrated both *in vitro* and *in vivo*.

## 2. Materials and Methods

Experimental procedures were carried out under the guidelines of the Monash University, Animal Ethics Committee, and conducted according to the International guidelines for Biomedical Research Involving Animals. All chemicals were sourced from Sigma (Castle Hill, Australia) unless otherwise stated.

### 2.1. Generation of Induced Pluripotent Stem (iPS) Cells from Adult Equine Fibroblasts

#### 2.1.1. Transfection, Isolation, and Culture of iPS Cells

Equine iPS cells were generated as previously reported [28]. Briefly, for VSVG pseudotyped retroviral production 3 × 10^6^ GP2 293 cells (Clontech; Scientifix, Cheltenham, Australia) were seeded in a 100 mm culture dish one day before transfection and incubated overnight at 37°C, 5% CO_2_. pMX-based retrovirus vectors encoding human DNA sequence of *OCT4, SOX2*, and* KLF4* were transfected into packaging cells (GP2 293) by FuGENE 6 transfection reagent (Roche, Castel Hill, Australia), and the media were replaced by fresh media on the following day. Viral supernatant was collected 48 and 72 hours later and filtered through a 0.45 *μ*m cellulose acetate filter. Viral supernatants were then mixed with polybrene to a final concentration of 8 ng/mL. Adult equine fibroblasts were plated one day prior to transduction at a density of 1 × 10^5^ cells per 100 mm dish. The cells were incubated overnight with the viral supernatant including equal contributions of the factors and 8 ng/mL polybrene. The following day, transduction process was performed similar to the first day. A *pMX-GFP* and no-vector dishes were provided as a positive and negative control, respectively. Transduced cells were then cultured in conventional medium containing *α*-minimum essential medium (*α*-MEM) with deoxyribonucleosides and ribonucleoside (Invitrogen, Mulgrave, Australia), supplemented with 2 mmol/mL glutamax (Gibco, Invitrogen, Mulgrave, Australia), 0.1% (v/v) Mercaptoethanol (Gibco), 1% (v/v) nonessential amino acid (NEAA) (Gibco), 1% (v/v) ITS (10 *μ*g/mL insulin, 5.5 *μ*g/mL 125 transferrin, 6.7 ng/mL selenium; Gibco), 5 ng/mL human LIF (Millipore, North Ryde, Australia), 10 ng/mL *β*FGF (Millipore), 10 ng/mL EGF (Invitrogen), 0.5% (v/v) penicillin-streptomycin (Gibco), and 20% (v/v) FBS. The medium was changed every other day to maintain cell proliferation. After 12 to 16 days of iPS induction, the best colonies based on equine ES cell-like colony's morphology were picked and manually passaged onto mouse embryonic fibroblasts (MEFs) inactivated with 4 *μ*g/mL of mitomycin C and plated in an organ culture dish. Colonies were manually cut into small clumps by insulin syringe needles and expanded on the freshly inactivated feeder layers to maintain the EiPS cell line. Seven cell lines were initially produced and maintained in culture, and one cell line was characterised in detail. The transduction efficiency of adult equine fibroblast was evaluated by expression of the *pMX-GFP* vector control, which was conducted in parallel with the iPS induction experiments. Seventy-two hours after *pMX-GFP* induction, cells were photographed under a fluorescence microscope, and the percentage of cells expressing GFP was quantified by flow cytometry. Reprogramming efficiency evaluated by correlation of *pMX-GFP* transduction efficiency with iPS cell colony numbers was established [17]. 

#### 2.1.2. FACS Analysis

Cells were incubated in incubator (37°C, 5% Co_2_) using 0.25% trypsin-EDTA (Invitrogen) for five min and dissociated through pipetting. After spinning at 400 g for 3 min, the pellet was resuspended and filtered through a 40 *μ*m cell strainer (BD Falcon) and analyzed by a BD FACSCanto Flow Cytometer (BD).

### 2.2. Characterization of Equine iPS Cell Lines

#### 2.2.1. Alkaline Phosphatase and Immunofluorescence Staining

Cells were fixed for 15 min in 4% (w/v) paraformaldehyde at room temperature and then stained. For alkaline phosphatase (ALP) activity, the cells were stained by histochemistry according to manufacturer's instructions using Alkaline Phosphatase Detection kit (Millipore). For OCT4 and NANOG staining, the cells were permeabilized in 0.2% Triton X-100 in 3%(v/v) goat serum in DPBS for 15 min. The cells were incubated with 3%(v/v) goat serum in DPBS at RT for 1 hr to block nonspecific binding of the primary antibodies and then incubated with primary antibodies raised against mouse anti-human SSEA1 (Millipore, MAB4301), mouse anti-human SSEA-4 (Millipore, MAB4304), mouse anti-human OCT4 (Santa Cruz, sc-5279) and rabbit anti-human Nanog (Abcam-ab21603) diluted at 1 : 100 in DPBS containing 3% (v/v) goat serum overnight at 4°C. The next day the dishes were washed with DPBS three times and incubated with secondary antibodies (diluted in DPBS 1 : 1000, Alexa Flour 594 or 488, Invitrogen) for 1 hr at RT. After three washes with DPBS, the cells were counterstained with 1 *μ*g/mL Hoechst 33342 in DPBS for 10 min at RT. Control cell lines were treated mouse ESD3 and human ES cells as well as negative control by omitting the primary antibodies (Supplemental Figures  1 and  2) (In Supplementary Material available on line at doi: 10.1155/2012/429160). Images were captured on an Olympus Ix71 microscope.

#### 2.2.2. RNA Extraction and RT-PCR Analysis of Gene Expression

Gene expression was analyzed by RT-PCR. Total RNA was extracted from harvested cell samples using Dynabeads mRNA DIRECT Micro Kit (Invitrogen) or using the RNeasy kit (Qiagen, Doncaster, Australia) according to the manufacturer's instructions. RNA concentrations were determined using the nanoDrop ND-1000 (NanoDrop Technology, Australia). The extracted RNA was treated by RQ1 DNase (Promega, South Sydney, Australia) to remove any contaminating genomic DNA. cDNA was generated using the superscript III enzyme as described before [30]. The first strand cDNA was further amplified by PCR using forward and reverse primers for specific genes. All samples were checked for *GAPDH* to verify the success of the RT reaction and then for other specific genes with individual primers. PCR amplification was performed in 50 *μ*L reaction containing 5 *μ*L DNA polymerase 10x reaction buffer, 3 *μ*L MgCl_2_ (25 mM), 1 *μ*L dNTP mixture (10 mM), 0.4 *μ*L GoTaq DNA Polymerase, 1 *μ*L (10 *μ*M) from each forward and reverse primer, 1 *μ*L sample and *μ*L Milli-Q water (Promega). The PCR was processed in a MyCycler Thermal Cycler and run for 35 cycles: denaturation (95°C, 45 s), annealing (55–56°C), and extension (72°C, 45 s) steps.

All PCR samples were analyzed by electrophoresis on a 2% (w/v) agarose gel. The sequence of primers used for PCR and the product size are listed in the Table 1.

#### 2.2.3. Chromosome Counts of Equine iPS Cell Lines

Chromosome counts were performed at P15 and P22. To estimate chromosome number, the cells were treated with 5-bromo-2-deoxyuridine (BrdU) overnight and then with Colcemid (Gibco) for a further 4 hours to suppress mitosis. After treating with TrypLE Express (Invitrogen) and hydrating in hypotonic KCL for 15 min, they were washed and fixed in methanol and acetic acid in a ratio of 3 : 1 and centrifuged. The fixation and centrifuge process were repeated three times. The fixed cell pellet was resuspended in 50 uL fixative and was dropped onto clean slides at RT. The slides were stained with a freshly made staining solution containing 3 mL of Leishman stain in 17 mL Gurrapostrophes buffer (Invitrogen) for 8 min. The Leishman stain was prepared by dissolving 2 g Leishman powder in 1 liter methanol. A coverslip was mounted on the slides with Histomount and slides viewed using a light microscope under oil immersion optics (Nikon C1) at 1000x magnification.

### 2.3. Differentiation Potential of Equine iPS Cell Line

#### 2.3.1. Embryoid Body Formation

Equine iPS cells colonies were mechanically dissociated into clumps with needles and cultured on Petri dishes in medium containing *α*-MEM with deoxyribonucleosides and ribonucleoside supplemented with glutamax (Gibco), mercaptoethanol (Gibco), nonessential amino acid (NEAA, Gibco), ITS (insulin, transferrin, selenium; Gibco), penicillin-streptomycin (Gibco), and FBS [30] at 39°C in a humidified gas environment of 5% CO_2_ in air. Culture medium was changed every 3 days. Samples from attached and nonattached EBs were collected at two weeks to check gene expression of ectodermal markers (*β*-tubulin III), endodermal markers (Gata4), and mesodermal markers (BMP4) (Table 1) by RT-PCR as described before.

#### 2.3.2. Teratoma Formation

Equine iPS colonies were dissociated into single cells and left on ice until preparation of mice for injection. Five-week-old male SCID mice were used for hind leg muscle injection of 2 × 10^6^ EiPS cells. All procedures were performed with sterile materials in a biological safety cabinet. They were then monitored for well-being and teratoma formation. A growth in the hind leg was visible after approximately 8–10 weeks after injection. Mice were humanely sacrificed; the tumor was dissected out, washed in DPBS, fixed in HistoChoic,e and embedded in paraffin for histological analysis. The samples were sectioned at 4 *μ*m thickness onto superfrost slides and allowed to dry overnight. After staining with hematoxylin and eosin, sections were observed using an Olympus Ix71 microscope. 

## 3. Freezing and Thawing

One hour before freezing the cells, a cryofreezing container containing isopropanol was equilibrated at 4°C. The colonies were dissociated into small clumps about 100 to 200 cells and collected into 15 mL falcon tube and washed by iPS cells medium and centrifuged for 3 min at 400 g. Supernatant was discarded, and clumps were resuspended in appropriate amount of EiPS cells medium. Freezing medium which consists of 80% FBS (JRH Bioscience, Australia) supplemented with 20% dimethyl sulphoxide (DMSO) was added to prepared 500 *μ*L suspension including 80–100 clumps of putative EiPS cells in iPS medium in a cryovial (Nunc, Thermo Fisher, Scoresby, Australia). Then the vials were initially frozen to −80°C overnight and then transferred to a LN_2_ tank at minus 196°C for long-term storage. The thawing process involved the placing of the cryovials containing the clumps of EiPS cells in a water bath at 37°C to be thawed, and cells were transferred to a 15 mL falcon tube, and then 10 mL iPS cells medium was slowly added. The cells were centrifuged for 3 min at 400 g, and then supernatant was discarded, the pallet was resuspended with EiPS medium, and clumps were implanted on fresh MEF in a culture dish using insulin syringe needle.

## 4. Results

### 4.1. Generation of Induced Pluripotent Stem (iPS) Cells from Adult Equine Fibroblasts

**Figure 1:**
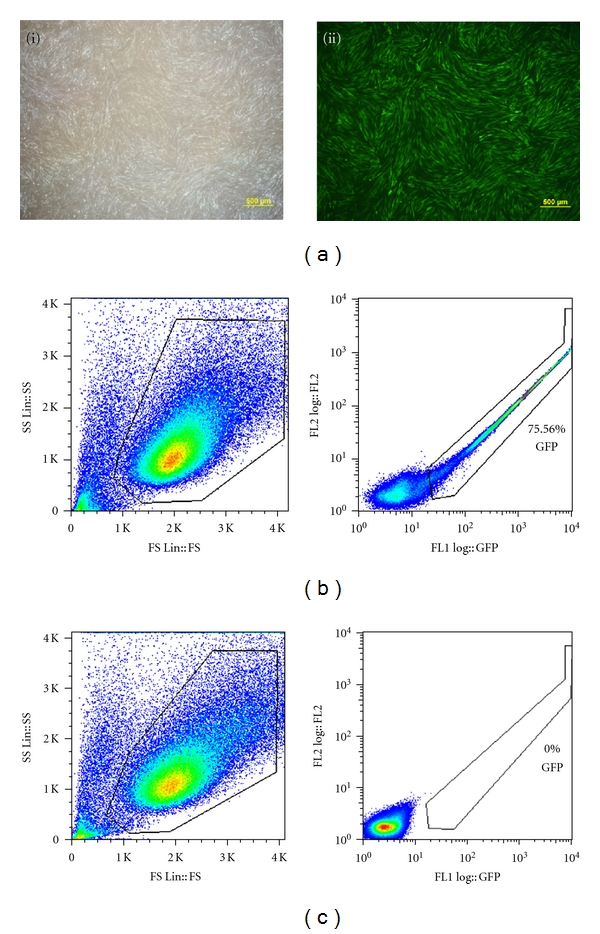
FACs analysis showing GFP fluorescence in adult equine fibroblasts following *pMX-GFP* viral transduction. (a) GFP fluorescence in AEFs following GP2293 mediated retroviral transduction, scale bar 200 *μ*M. (i) Bright filed. (ii) Green filter. FACs profile of GFP fluorescence, (b) Retroviral transduction using GP2 293 packaging cell. (c) Control EAFs.

After two rounds of repeated transduction with the three factors (*OCT4, SOX2*, and *Klf4*) into adult equine fibroblast, we achieved a transduction efficiency of greater than 60% on day two postinfection using *pMX-GFP* control plasmid, while negative control showed no *GFP*-marked cells (Figures 1(b) and 1(c)).

**Figure 2:**
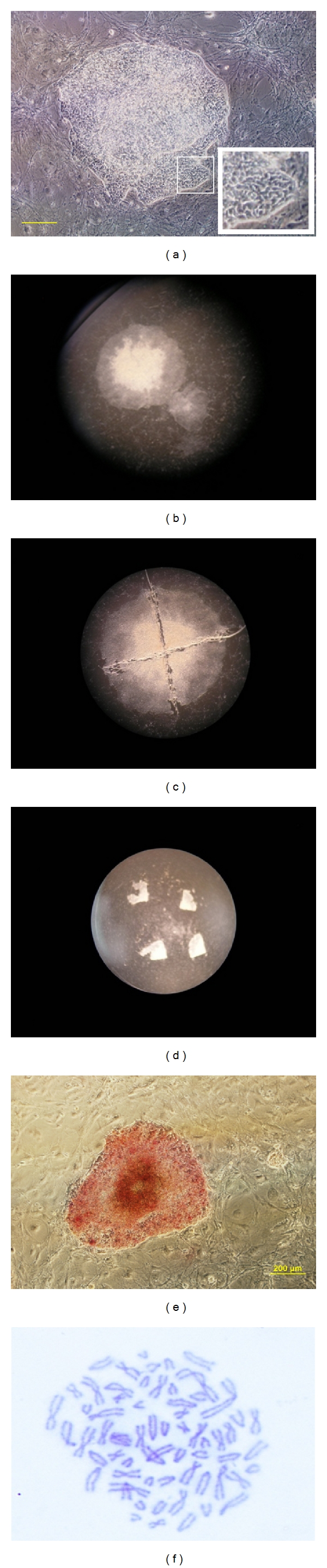
Generation of EiPS cells. (a) Morphology of EiPS single colony growing from individual fibroblast. (b) Typical colony of EiPS cells on the MEF and colony selection. (c) Cutting individual colony for manual passaging. (d) Passaged pieces on the MEF. (e) Alkaline phosphatase activity of EiPS cells scale bar 200 *μ*M. (f) Chromosome spread of EiPS cells.

iPS cell colonies first appeared on the day 8–10 postinfection with the dome-like and tightly packed structure. They became large enough at around day 16 to be picked and expanded. Colonies were isolated mechanically and transferred onto prepared culture dishes containing MEF layer and equine ES cell medium (Figure 2).

### 4.2. Characterization of Equine iPS Cell Line

**Figure 3:**
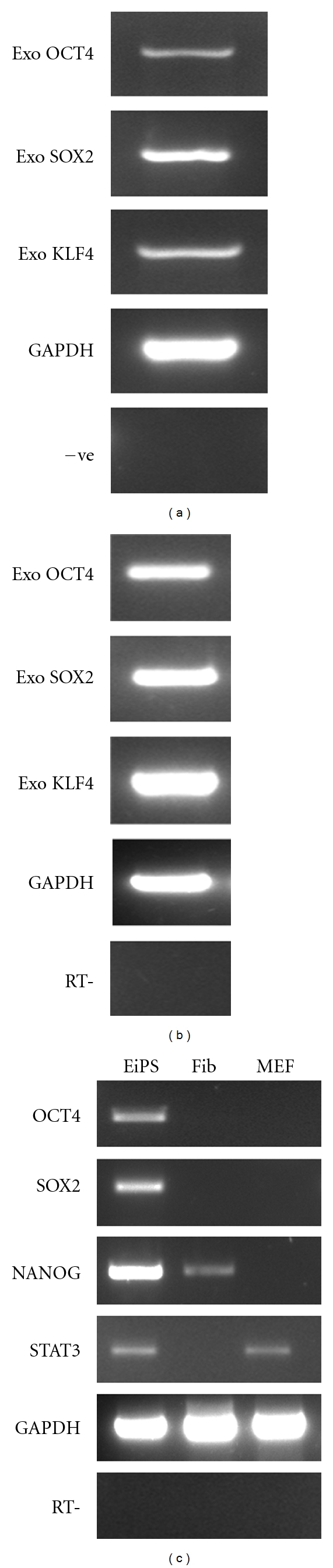
Gene expressions profile of EiPS cells and genomic DNA analysis. (a) Genomic PCR confirming the integration of the four transgenes. (b) Gene expression of exogenous reprogramming factors. (c) Gene expression profile of the EiPS cells compared to the parental EAFs and MEF as feeder cells.

**Figure 4:**
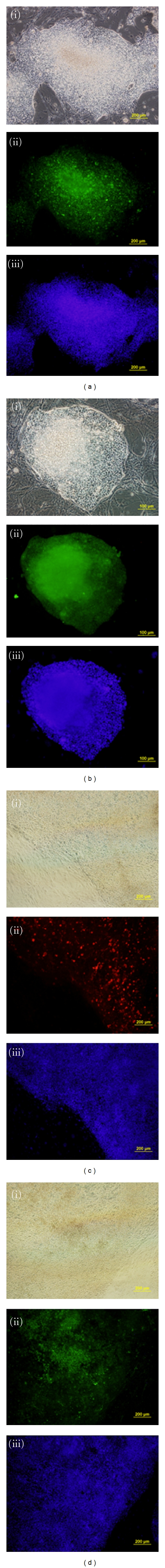
Immunoflourescence staining of pluripotent markers in EiPS cells. Immunostaining of EiPS cells for (a) *OCT4*, (b) *NANOG*, (c) *SSEA1*, and (d) *SSEA4*, counterstained with DAPI, scale bar 200 *μ*M.

EiPS cells had a low cytoplasm to nuclear ratio and formed colonies to those observed in cattle [28]. The cell line was characterized by molecular analysis. The integration of reprogramming transgenes into the genome of the cells was confirmed by gDNA PCR analysis and expression of exogenous factor examined at passage 24 (Figures 3(a) and 3(b)). RT-PCR analysis showed mRNA expression of key pluripotent markers including *OCT4, SOX2, NANOG*, and *STAT3* (Figure 3(c)). Some expression of Nanog was detected in equine fibroblasts, and STAT3 was also detected in the mouse embryonic feeder cells using the primer pairs. The cell line expressed a high level of alkaline phosphatase activity (Figure 2(e)). They were positive for protein expression of OCT4, NANOG, SSEA1, and SSEA4 as determined by immunofluorescent staining (Figure 4). Moreover, chromosome spreads revealed a normal diploid chromosome count of 64 in metaphase spreads at passage 15 (data not shown) and 22 (Figure 2(f)). More than 90% of frozen EiPS cells clumps were recovered after thawing and formed colonies after implanting on fresh MEF feeder layer. Thawed cell lines survived and were maintained for more than four passages without losing iPS cell morphology.

#### 4.2.1. Differentiation Potential of Equine iPS Cells

**Figure 5:**
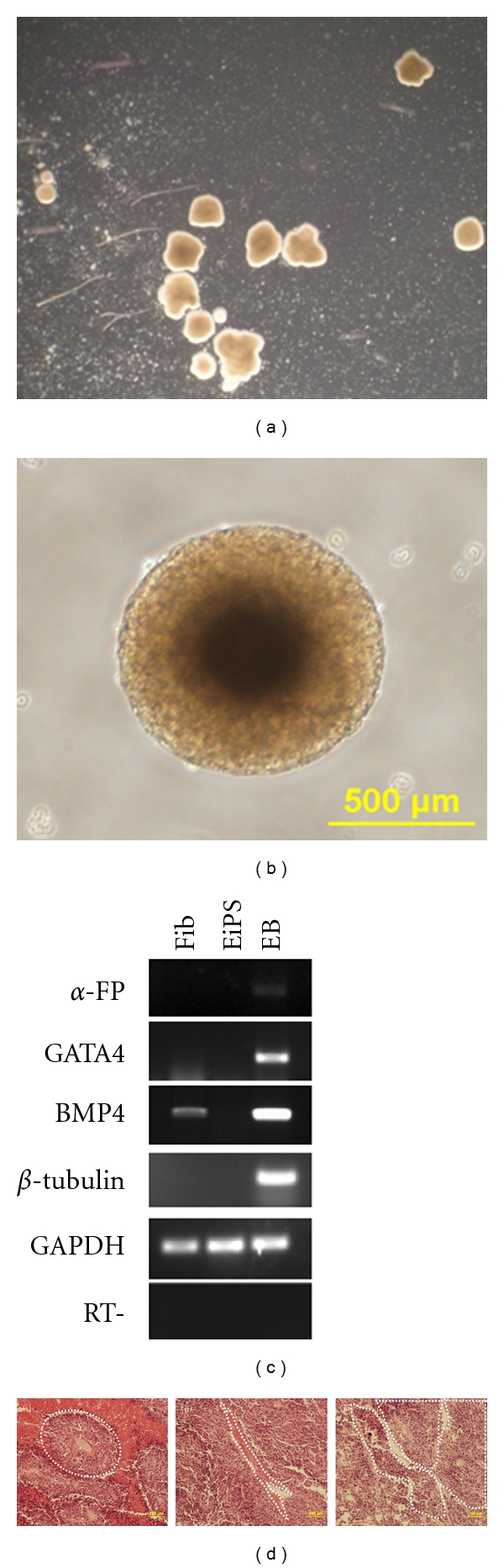
Differentiation potential of EiPS cells. (a) Embryoid body formation of EiPS cells grown in suspension medium in the absence of LIF. (b) Single EB, scale bar 500 *μ*M. (c) Gene expression profile of EiPS cells following differentiation of embryoid body. (d) Histology of differentiated tissues found in the hind leg muscle of SCID mice following injection of EiPS cells included (i) endodermal differentiation, (ii) mesodermal differentiation, (iii) ectodermal (neuroblastic) differentiation, scale bar 100 *μ*M.

The EiPS cells formed embryoid bodies after 5 days in suspension culture, after which they were transferred to gelatin-coated dishes to attach and develop outgrowths (Figures 5(a) and 5(b)). RT-PCR results demonstrated mRNA expression of genes representative of the three embryonic germ layers [11, 12], endoderm (*α*-fetoprotein), mesoderm (*Gata4 and BMP4*), and ectoderm (**β*-tubulinIII*) (Figure 5(c)). Equine iPS cells formed teratomas 8 to 10 weeks after injection containing cells of the three embryonic germ layers: endoderm (vessels), mesodermal cells (muscle), and ectoderm (epidermal cells) (Figure 5(d)).

## 5. Discussion

Due to similarity in size, physiology, and immunology, large animals are better models for human genetic or acquired diseases compared with rodents. In addition, they have a longer life span and have a heterogeneous genetic background which is similar to humans and unlike rodents; therefore, they can provide a good model for long-term experiments. Also about 95 equine genetic diseases share a high homology with human genetic defects [13]. Furthermore, equine can be an appropriate model for human diseases such as osteoarthritis as well as a model for musculoskeletal injuries as there are common features of the athletic injuries in human and equine. Limited capability for full functional repair of musculoskeletal injuries has limited treatments outcomes [1]. Joint injuries and related illnesses cost an estimated US$6.5 billion annually for the equine race industry [13].

MSCs, ESCs, and iPS cells are options for research and therapeutic applications regarding musculoskeletal injuries. Compared with MSCs and ESCs, iPS cells are better as they provide pluripotent cells that can be immunocompatible to the recipient. There is one report on induction of pluripotency in equine [29], using the Yamanaka cocktail (OSKM) to generate iPS cells from fetal cells. In this study we report the generation of equine iPS cells from adult cells and without the use of the protooncogene c-MYC which opens the door for autologous transplantation in cartilage and tendon injury models. Similar to the finding of Nagy and colleagues the equine iPS cells generated required continuous expression of the transgenes to maintain pluripotency. Apart from one report in sheep [21], iPS cells generated in other domestic species have shown similar traits [12, 18, 24, 25, 28], suggesting that maintenance of pluripotency largely depends on the expression of the reprogramming transgenes.

We established the equine iPS cell line which proliferated in culture beyond 27 passages. The cells maintained ESC characteristics and expressed pluripotent markers including alkaline phosphatase activity and expression of pluripotency markers OCT4 and NANOG. Furthermore, the cells stained positively for SSEA1 similar to mouse pluripotent cells; as well as SSEA4 which is expressed on human pluripotent cells, similar findings have been reported in equine ES [11, 12] and iPS cells [29]. The EiPS cells expressed pluripotency genes *OCT4, SOX2, NANOG*, and *STAT3* by RT-PCR. The EiPS cells showed differentiation potential *in vitro *by EB formation and expressing genes indicative of the three embryonic germ layers. Some of the discrepancies in the markers are due to the difficulties in characterizing pluripotency in the horse as there is a lack of reliable pluripotency markers [1] and lack of suitable antibodies raised against equine cells for immunocytochemical analyses [29]. Therefore, *in vivo *differentiation by teratoma formation was used as further evidence of pluripotential of the cells as has been routinely conducted for iPS cells from most domestic species.

In summary, our findings indicate that adult equine fibroblast can be reprogrammed into pluripotent state via the retroviral delivery of transcription factors, *OCT4, SOX2*, and* KLF4*. The generated iPS cells are pluripotent as shown by expression of pluripotent markers and have capability to differentiate into cell types indicative of the three embryonic germ layers both *in vitro* and *in vivo.*

## Supplementary Material

Immunofluorescence staining of control cell lines mouse ESD3 and human ES cells with SSEA1, SSEA4, Oct4 and Nanog. Negative control by omitting the primary antibodies. Images were captured on an Olympus Ix71 microscope.

## Figures and Tables

**Table 1:** List of primers used for RT-PCR.

Markers	Primer F	Primer R	References
* GAPDH*	GATTCCACCCATGGCAAGTTCCATGGCAC	GCATCGAAGGTGGAAGAGTGGGTGTCACT	
*OCT4*	TCT TTC CAC CAG GCC CCC GGC TC	TGC GGG CGG ACA TGG GGA GAT CC	
*NANOG*	TCA AGG ACA GGT TTC AGA AGC A	GCT GGG ATA CTC CAC TGG TG	
*SOX2*	GGT TAC CTC TTC CTC CCA CTC CAG	TTG CCT TAA ACA AGA CCA CGA AA	
*STAT-3 *	TCTGGCTAGACAATATCATCGACCTT	TTATTTCCAAACTGCATCAATGAATCT	Li et al. [12]
*β-Tubulin III*	CAGAGCAAGAACAGCAGCTACTT	GTGAACTCCATCTCGTCCATGCCCTC	Li et al. [12]
*GATA-4 *	CTCTGGAGGCGAGATGGGACGGG	GAGCGGTCATGTAGAGGCCGGCAGGCATT	Li et al. [12]
**α*-Fetoprotein *	CTTACACAAAGAAAGCCCCTCAAC	AAACTCCCAAAGCAGCACGAG	Li et al. [12]
*BMP4*	TCGTTACCTCAAGGGAGTGG	GGCTTTGGGGATACTGGAAT	Pashaiasl et al. [30]

*OCT4* and *SOX2 *primers were based on primers specific for* Homo sapiens* primers. The sequences of these genes were blasted against horse nucleotide sequences that have 95% and 94% coverage with the coding sequence of Equus caballus. *BMP4 *primers were designed on a bovine sequence that has 91% coverage with the coding sequence of Equallus equa. *STAT3, GATA4, β-tubulin III*, and **α*-fetoprotein *primers have been applied by Li et al. [12].
