# Supplementary figures and images for: Induction of Pluripotency in Adult Equine Fibroblasts without c-MYC
Source: Stem Cells Int. 2012 Mar 19;2012:429160. doi: 10.1155/2012/429160 (PMC3328202; doi:10.1155/2012/429160)

Figure - 1

A

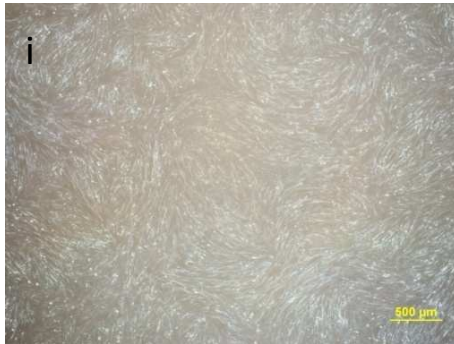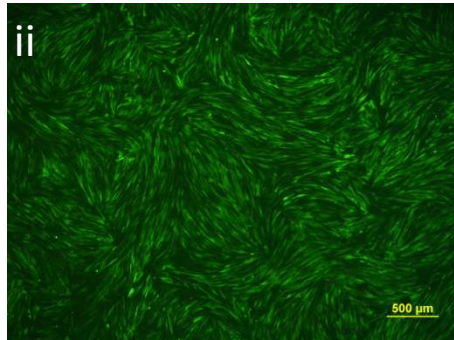

B

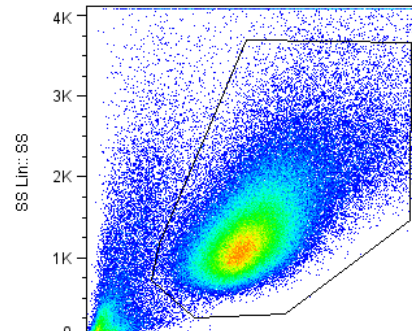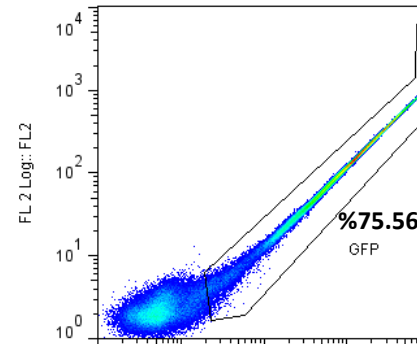

C

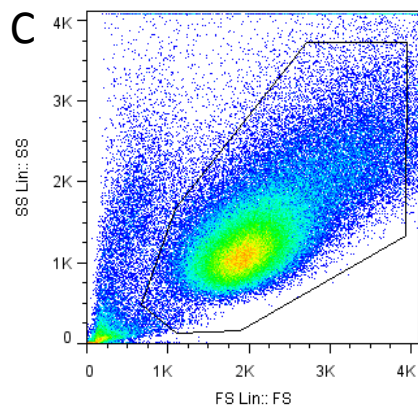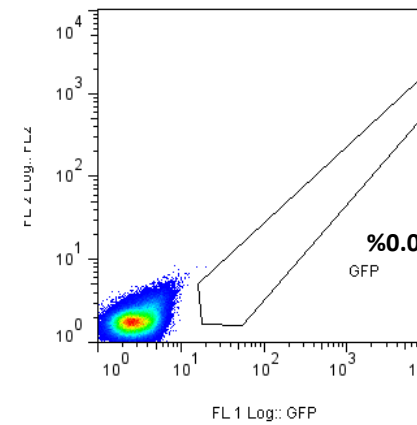

Figure - 2

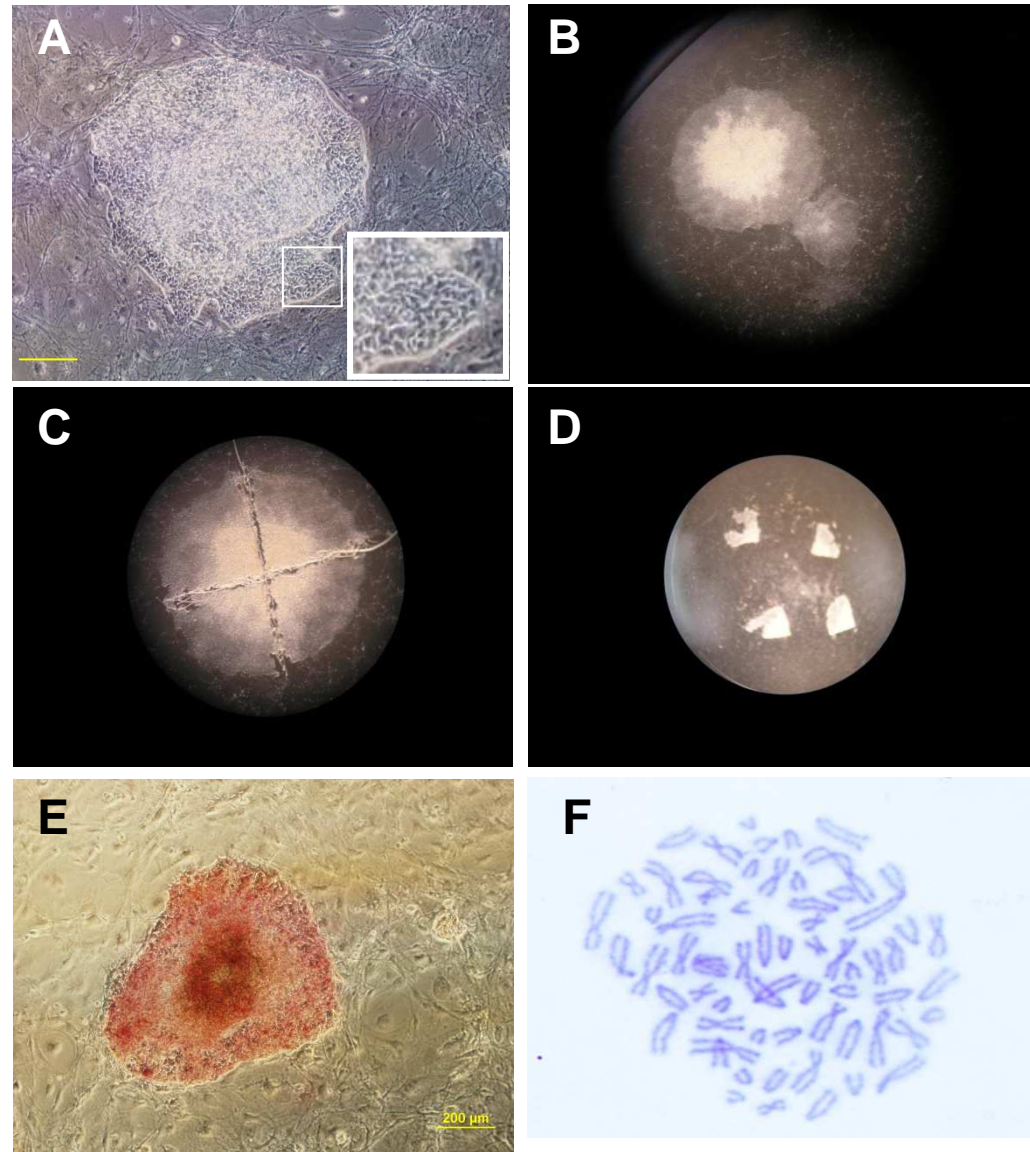

Figure - 3

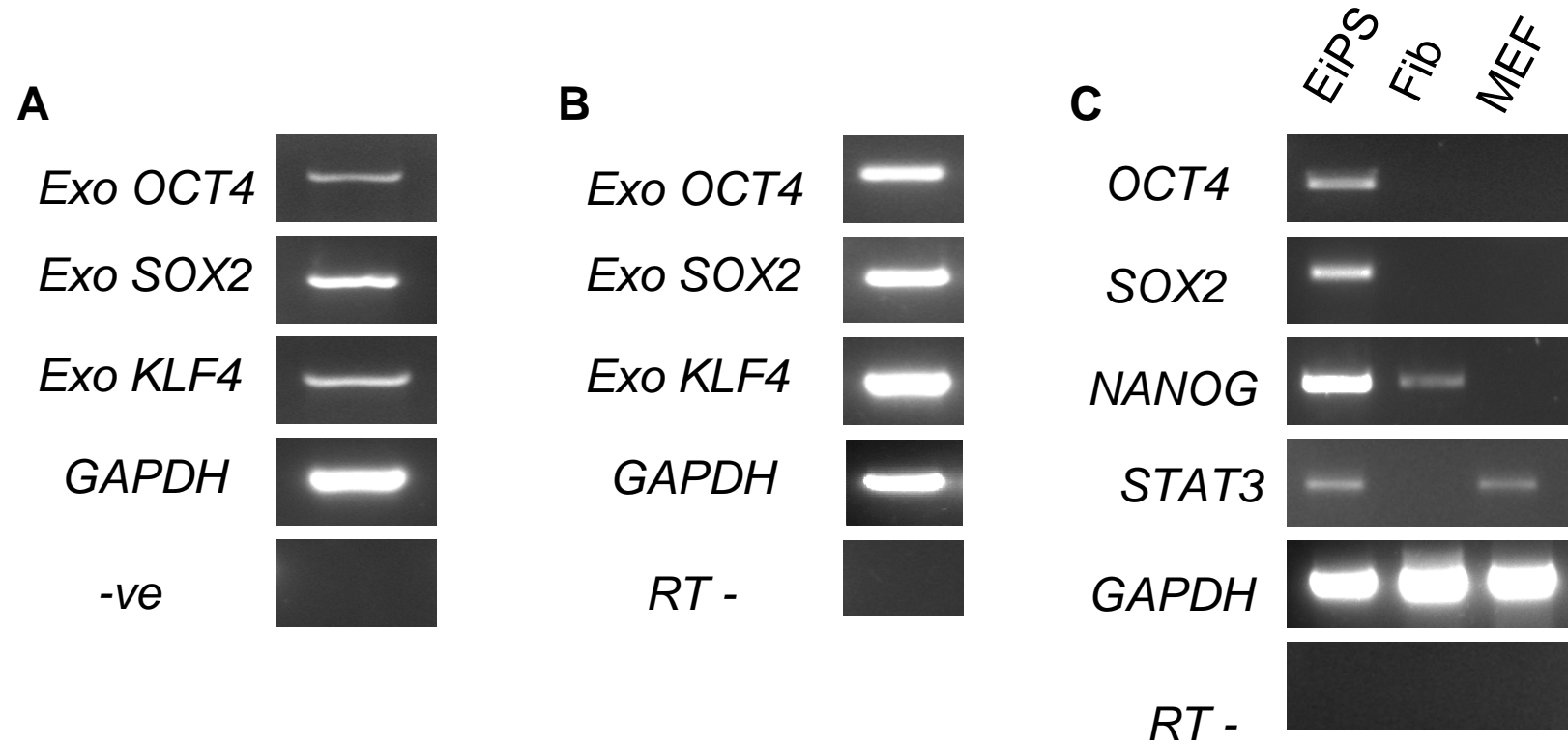

Figure - 4

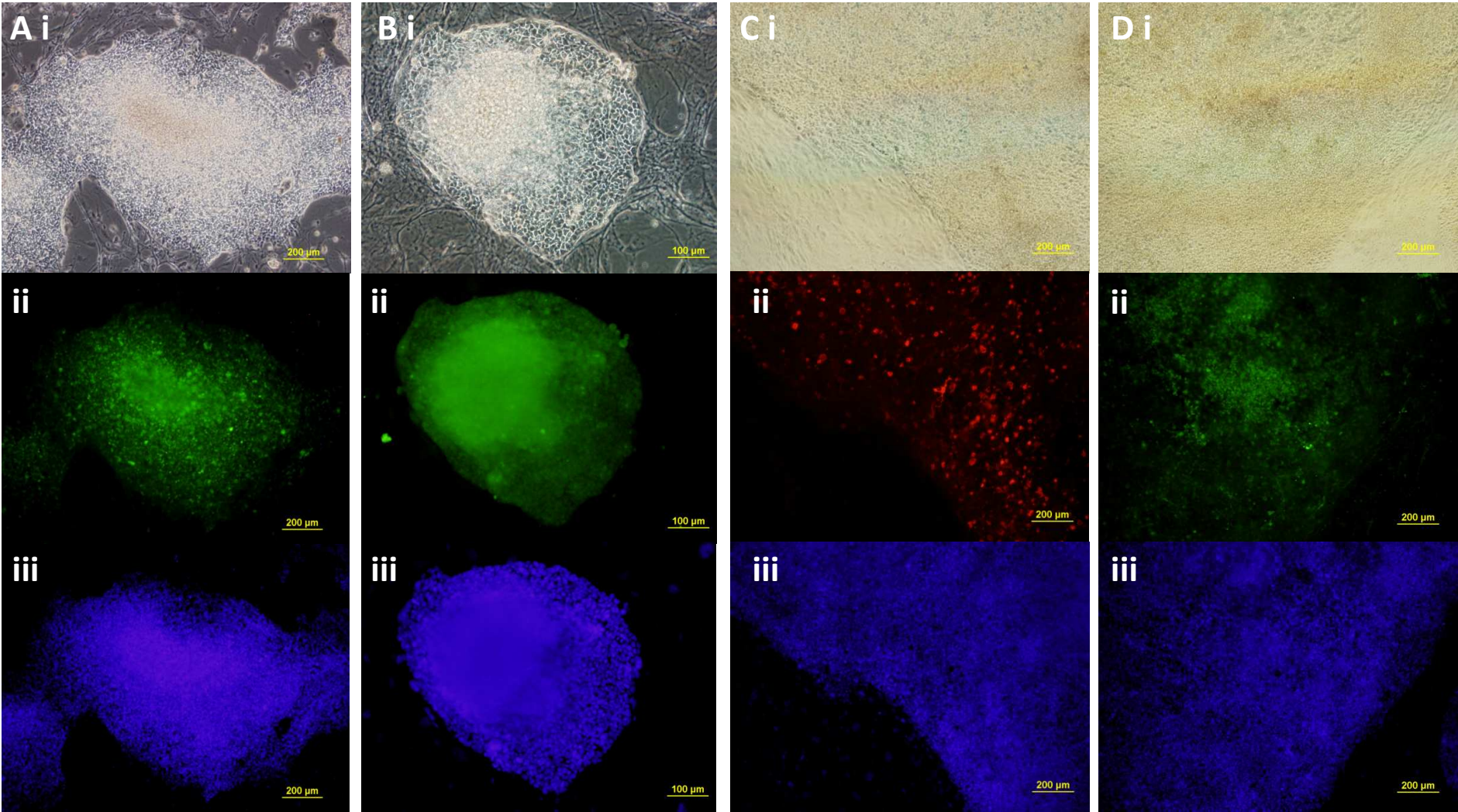

Figure - 5

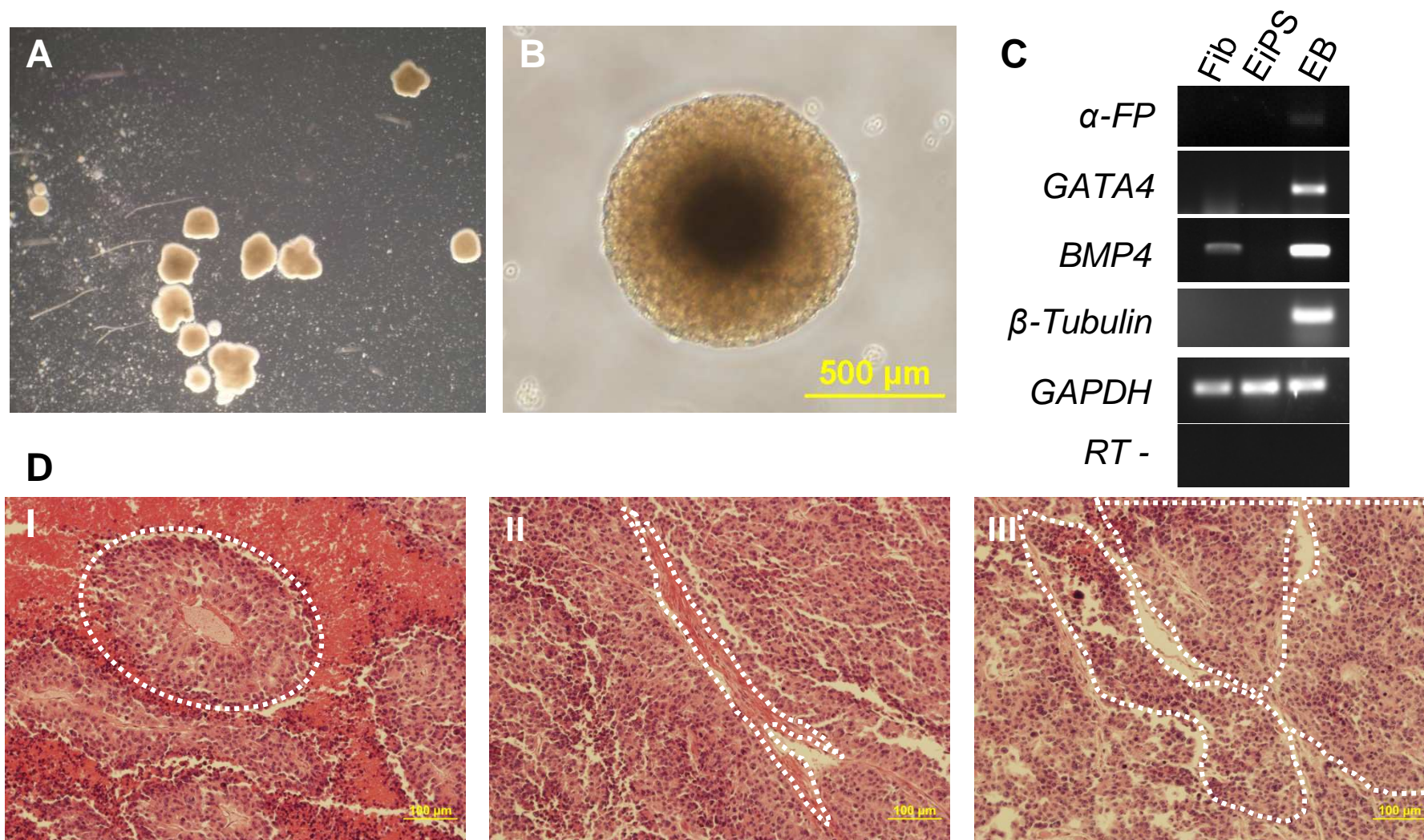

Supplemental Figure 1

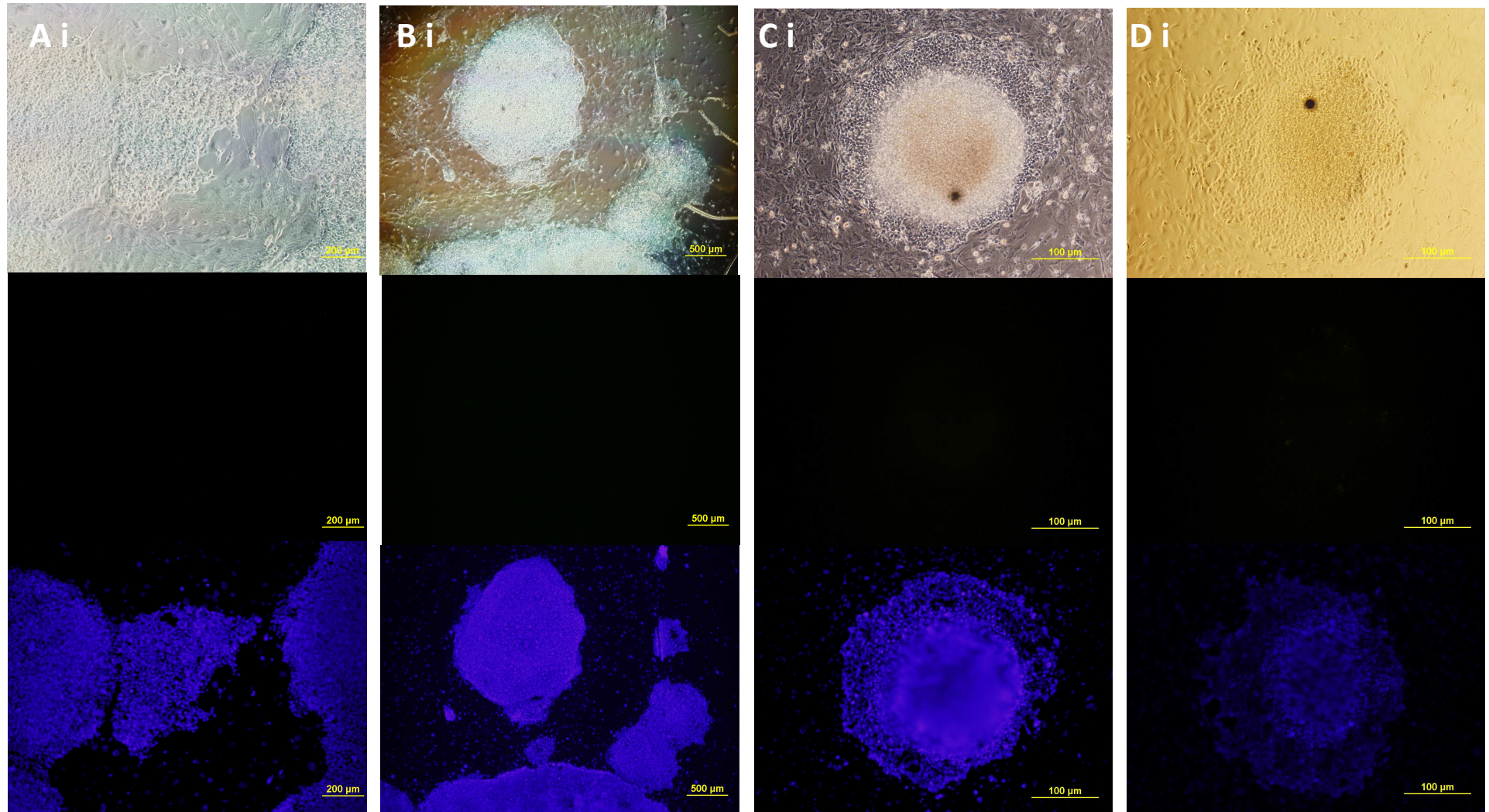

Supplemental Figure 2

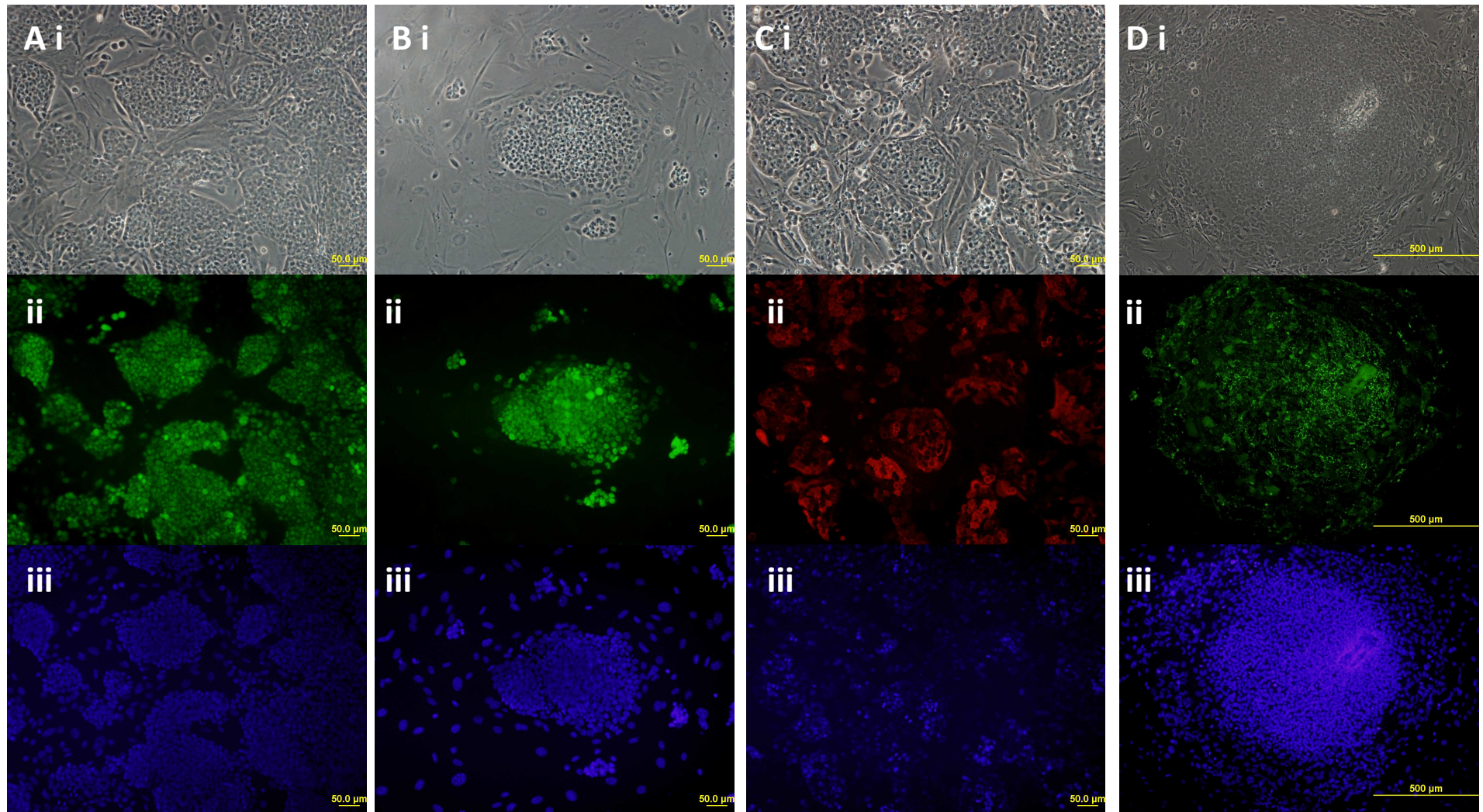

Supplement: Supplementary file 1 — Immunofluorescence staining of control cell lines mouse ESD3 and human ES cells with SSEA1, SSEA4, Oct4 and Nanog. Negative control by omitting the primary antibodies. Images were captured on an Olympus Ix71 microscope. [file 429160.f1.pdf]
